# FGF7 mitigates airway inflammation and epithelial injury in cigarette smoke-induced COPD model

**DOI:** 10.3389/fimmu.2026.1815550

**Published:** 2026-06-08

**Authors:** Xinji Gong, Jingwen Li, Haitao Wang, Xi Luo, Xinying Hu, Jie Shen, Wenting Jia, Qiufeng Wan, Shareli Caikai, Zhijin Guo, Fang Yan, Ying Zhang, Feng Sun, Sicheng Xu

**Affiliations:** Respiratory Intensive Care Unit, Department of Pulmonary and Critical Care Medicine, Center of Respiratory Medicine, The First Affiliated Hospital of Xinjiang Medical University, Urumqi, China

**Keywords:** airway inflammation, chronic obstructive pulmonary disease, epithelial injury, ERK1/2 signaling pathway, FGF7

## Abstract

**Background:**

Chronic obstructive pulmonary disease (COPD) is a chronic inflammatory airway disease marked by persistent airway limitation. Airway epithelial injury is pivotal in the onset and progression of COPD. Fibroblast growth factor 7 (FGF7) has been reported to promote alveolar epithelial regeneration following lung injury. Nonetheless, how FGF7 protects epithelial cells against cigarette smoke (CS)-induced airway injury remained unclear.

**Methods:**

FGF7 levels were analyzed in human lung tissues (n=32; 17 COPD and 15 control subjects) and serum samples (n=71; 51 COPD and 20 control subjects) using immunohistochemistry, RT-qPCR, Western blot, and ELISA. In a COPD rat model subjected to CS exposure for 12 weeks, the impact of intratracheal administration of AAV-FGF7 or AAV-shFGF7 on lung function was assessed by lung ventilation and histology, expression levels of cytokines, and other signaling molecules. In the CSE-injured 16HBE cell model, recombinant FGF7 was applied with or without SB202190 (a p38 inhibitor), LY294002 (a PI3K inhibitor), or AG1478 (an EGFR inhibitor) to evaluate cell viability, migratory capacity, cytokine production, and activation of corresponding signaling pathways.

**Results:**

COPD patients demonstrated an increased level of FGF7 in lung tissues, while serum FGF7 levels decreased. In the rat model, CS exposure led to heightened airway inflammation, collagen deposition, and elevated cytokine levels in bronchoalveolar lavage fluid (BALF). Transfection of AAV-FGF7 resulted in improvements in MVb/PIFb/EF50, a reduction in inflammation and peribronchial fibrosis, and decreased levels of IL-1β, IL-6, TNF-α, TGF-β1, and ET-1 in BALF. Correspondingly, AAV-shFGF7 aggravated these pathological effects. FGF7 was found to enhance the phosphorylation of ADAM17 and EGFR, as well as ERK1/2, p38, and AKT, whereas knockdown of FGF7 inhibited the activation of these signaling pathways. In 16HBE cells, FGF7 was observed to restore cell viability and migration, suppress cytokine release, and activate ADAM17, EGFR, and ERK1/2, and these effects were diminished by the blockade of p38, PI3K, or EGFR.

**Conclusions:**

FGF7 is specifically upregulated in the lung tissues of COPD patients. It mitigates CS-induced airway epithelial damage and inflammation through the ADAM17-dependent EGFR-ERK1/2 axis, p38, and PI3K/AKT pathways. FGF7 therefore emerges as a promising therapeutic target for interventions aimed at preventing airway remodeling in COPD.

## 1 Introduction

Chronic obstructive pulmonary disease (COPD) is a heterogeneous inflammatory airway disease characterized by sustained airway limitation (1). It ranks the third leading cause of mortality globally, responsible for approximately 3 million deaths each year and affecting over 400 million individuals worldwide (2). Pathologically, the progression of COPD is characterized by a series of alterations in airway pathophysiology, including chronic inflammation, airway remodeling, and irreversible tissue destruction.

COPD arises from a confluence of multiple risk factors, of which cigarette smoking is the most prevailing one (2). Exposure to cigarette smoke (CS) compromises the integrity of the airway epithelial barrier, disrupts epithelial junctions, and contributes to aberrant repair of airway epithelium (3). CS-induced epithelium damage serves as both a source of persistent inflammation and a principal driver of progressive lung dysfunction and tissue damage in COPD.

Increasing evidence suggests that genetic predisposition also accounts for a significant proportion of COPD risk (4, 5). Identifying susceptible genetic background can enhance our understanding of the complex pathological mechanisms underlying COPD and facilitate the identification of novel pharmacologically targetable pathways. In a prior case-control study, we identified fibroblast growth factor 7 (FGF7) as a gene associated with COPD susceptibility (6). FGF7, also referred to as keratinocyte growth factor (KGF), is an epithelial cell-specific growth factor (7, 8). It plays crucial roles in epithelial cell proliferation, differentiation, and repair. In the context of lung health, FGF7 is critical for bolstering pulmonary innate immune defense. It has been demonstrated that FGF-deficient mice exhibited delayed bacterial clearance and diminished antimicrobial activity, which can be reversed by the administration of recombinant human FGF7 (9). Moreover, FGF7 plays a pivotal role in the repair and regeneration of lung tissue post-injury. In models of acute lung injury, FGF7 has been shown to upregulate surfactant proteins and enhance macrophage phagocytic activity, both of which are vital for resolving inflammation and repairing damaged tissues (10). Additionally, FGF7 has been implicated in maintaining the renewal capacity of the alveolar epithelial stem cell, modulated by the STAT3–BDNF–TrkB axis (11). Its involvement in the proliferation and differentiation of alveolar epithelial type II cells further underscores its importance in lung tissue homeostasis and repair (12).

Despite the documented functions of FGF7 in lung injury models, its protective role in chronic inflammatory diseases such as COPD remains insufficiently understood. In this study, we observed an upregulation of FGF7 in lung tissue samples of COPD patients. In both CS-induced COPD rat models and cell models, ectopic expression of FGF7 significantly improved lung function, reduced airway inflammation, and mitigated tissue remodeling. Conversely, knockdown of FGF7 exacerbated these pathological effects, suggesting a protective role of FGF7 in CS-induced airway epithelial cell damage. As a locally expressed epithelium protective factor, FGF7 exerts its anti-inflammatory and reparative functions through the ADAM17-dependent EGFR/ERK signaling pathway. This study elucidates the role of FGF7 in CS-induced airway inflammation and epithelial injury, and identifies this pathway as a potential target for COPD intervention.

## 2 Materials and Methods

### 2.1 Human samples

Lung tissue specimens of both COPD and non-COPD individuals were obtained from patients undergoing lobectomy due to suspected lung tumor nodules at the First Affiliated Hospital of Xinjiang Medical University from November 2016 to January 2020 (Urumqi, China). All participants provided written informed consent prior to enrollment. The patient enrollment and research protocol were approved by the Ethics Committee of the First Affiliated Hospital of Xinjiang Medical University (Approval No. K202105-06). Prior to surgery, each participant underwent a standardized pulmonary function test (Master Screen, Jarger, German). The severity of COPD was classified according to the Global Initiative for Chronic Obstructive Lung Disease (GOLD) guidelines, with classification criteria as follows: mild group, FEV1/FVC<70% and FEV1 ≥ 80% predicted; moderate group, FEV1/FVC < 70% and 50% ≤ FEV1 < 80% predicted; severe group, FEV1/FVC < 70% and 30% ≤ FEV1 < 50% predicted. Lung tissue samples were excised from the lung parenchyma at a distance of >2 cm from the tumor/nodule margins. All tissue sections were reviewed by experienced pathologists to ensure that the areas used in the study were devoid of tumor invasion or localized lesions. Peripheral venous blood samples were collected from the same cohorts following standard procedures.

### 2.2 Animal studies

Male Wistar rats (SPF grade; 8–9 weeks old; 250 ± 20 g; n=60) were supplied by the Xinjiang Medical University Animal Experimental Center and were maintained under standard pathogen-free conditions before randomization. All animal experiments were conducted in compliance with the institutional guidelines and were approved by the ethics committee at the center (Approval No. IACUC-20160218-035).

To induce chronic smoke injury, rats were subjected to mainstream smoke exposure from commercial cigarettes (Chinese Baisha) for a duration of 4 hours/day over a 12-week period (13–15). The exposure was conducted using a custom-designed glass fumigation chamber (70 cm × 50 cm × 30 cm). A total of 12 cigarettes were combusted per hour, with the process conducted twice daily and a 2-hour interval between sessions. Control groups were subjected to identical handling procedures without smoke exposure.

For airway-targeted interventions, the rats were randomized into six groups (n=10 each): control, model, model+AAV-NC, model+AAV-FGF7, model+AAV-shRNA-NC, and model+AAV-shFGF7. The shRNA lentiviral particles comprised three distinct shRNA sequences specifically targeting FGF7. The sequences for FGF7 shRNA and NC sh-RNA were as follows. shFGF7-1:GCAATGAGGATTGCAACTTCA; shFGF7-2: GCTACAACATCATGGAAATCA; shFGF7-3: GCGACACACGAGAAGTTATGA; shNC: TTCTCCGAACGTGTCACGT. All recombinant adeno-associated virus vectors (AAV) were purchased from GeneChem(Shanghai, China). Virus-treated groups received a 50 μL intratracheal instillation of adenovirus at 5×10^11^ vg/mL throughout the 12-week exposure period, while control groups received an equivalent volume of saline parallelly.

Following the 12-week exposure, all animals underwent lung function testing (Buxco system, USA). Serum samples were collected from the retro-orbital veins using heparin-free capillary tubes after anesthesia with isoflurane. Pulmonary function tests in rats were performed using the animal lung function measurement system in compliance with the standard operating protocol (Buxco, PFT Maneuvers). Following these assessments, the rats were anesthetized via intraperitoneal injection and then subsequently euthanized. After euthanization, the left lung was lavaged with 1×PBS buffer (Maixin, Fuzhou, China) to collect BALF samples. The BALF was immediately centrifuged at 700×g for 10 minutes to separate the cell pellet and supernatant for cell counting and biochemical analysis. The right lung was dissected into three segments: one segment was fixed with 4% neutral-buffered formalin for histological and immunohistochemical analysis, while the remaining two segments were snap-frozen in liquid nitrogen and stored at -80°C for subsequent RNA and protein extraction.

### 2.3 Hematoxylin and eosin and Masson staining

HE and Masson staining were conducted in accordance with our published work (16). In brief, lung tissue samples were fixed in 4% neutral-buffered formalin for 48 hours, embedded in paraffin, and sectioned into 4 μm slices for HE staining. For the assessment of lung morphometry, the mean linear intercept (MLI) was measured using ImageJ software on HE-stained sections to quantify the average size of the alveolar spaces. Briefly, a grid of equidistant horizontal and vertical lines was superimposed on five randomly selected non-overlapping fields per slide. The MLI was calculated by dividing the total length of the grid lines by the total number of intercepts with the alveolar septa. To evaluate collagen deposition in the airway wall, sections were baked at 65°C for 2 hours and stained with Masson’s trichrome (D026-1, Nanjing Jiancheng) following the manufacturer’s protocol.

### 2.4 Immunohistochemistry

IHC was performed as previously described (16). Human lung tissues were fixed in 4% neutral-buffered formalin for 48 hours, embedded in paraffin, and sectioned into 4 μm slices. IHC detected FGF7/KGF expression, sections were deparaffinized and rehydrated, and then, endogenous peroxidase was quenched with 3% aqueous hydrogen peroxide for 10 min. Tissue samples were incubated overnight at 4°C with a primary antibody against KGF (1:100; ab90259 or ab131162, Abcam). After washing, the samples were incubated with goat anti-rabbit IgG-HRP (1:5000, ab205718, Abcam) for 20 minutes at room temperature. DAB (Maixin, Fuzhou, China) was used for visualization. Slides were scanned using an optical microscope(E200, Nikon, China)and analyzed with Image J based on 5 randomly selected regions. For each section, 5 fields were randomly chosen for observation under a 40x magnification. A semi-quantitative comprehensive scoring method was used to evaluate the alveolar and airway epithelium based on staining intensity: negative staining was assigned a score of 0, light yellow staining a score of 1, light brown staining a score of 2, and dark brown staining a score of 3. Additionally, the percentage of positive cells, defined as those with fully stained cytoplasm, was recorded. The final score was calculated as the product of the percentage of positive cells and the staining intensity score. The scoring criteria for the alveolar epithelium were as follows: product = 0 was 0 points (negative), 1 ≤ product < 60 was 1 point (weakly positive), 60 ≤ product < 180 was 2 points (moderately positive), and product ≥ 180 was 3 points (strongly positive). The scoring criteria for airway epithelium were: the product = 0 was 0 points (negative), 1 ≤ product < 120 was 1 point (weakly positive), and product ≥ 120 was 2 points (moderately positive).

### 2.5 Cell culture

16HBE human bronchial epithelial cells (Fenghui Biotechnology, Hunan, China) were cultured in DMEM supplemented with 10% fetal bovine serum(Suzhou Excell Bio, China), penicillin-streptomycin solution (GIBCO, China) at 37°C, 5% CO_2_. To induce inflammation and epithelial injury in 16HBE cell model, 5% of CSE was added to the cell culture for 24 hours. Concurrently, recombinant FGF7 (R&D, 20 ng/ml) was introduced into the cell culture 24 hours before the collection of cells and cell supernatant. Specific inhibitors, including SB202190 (Selleck, 10 μM), LY29400 (Selleck, 10 μM), and AG1478 (MCE, 10 μM) were used to pretreat the cells for 1 hour before the addition of CSE and rFGF7.

### 2.6 Cigarette smoke extract

The smoke from two Baisha cigarettes was pumped into 20 mL of serum-free medium (Procell, China), subsequently filtered through a 0.22 μm membrane (Millipore), and adjusted to pH 7.4. The medium containing CSE was diluted to OD305 = 1.0 to generate a 100% CSE stock solution. Experimental concentrations (1-20%) were prepared by further diluting the stock solution with culture medium.

### 2.7 Cell proliferation assay

The proliferation of 16HBE cells was measured using the CCK-8 kit (TransGen, China) according to the manufacturer’s instructions. Cells were seeded at a density of 5×10³ cells/well in 96-well plates and cultured for 24 hours. Following CSE treatment, cells were incubated with 100 μL of CCK-8 solution for 1 hour at 37°C. The absorbance at 450 nm was measured with a microplate reader.

### 2.8 Cell migration assay

The scratch wound healing assay was conducted to evaluate the migratory capacity of 16HBE cells. A single-cell suspension with a density of 5×10^5^/ml was prepared using complete medium. 70 μl of the single-cell suspension was added to each well. In the two central wells of the petri dish, after the cells covered the Insert area, the Insert was removed with forceps to create a 500 μm-wide scratch. Then, 1 ml of the serum-free medium was added to intervene the cells for 24 hours. Images were taken at the same location every 8 hours. The scratch areas were quantified using Image J.

### 2.9 Enzyme-linked immunosorbent assay

IL-1β, IL-6, TNF-α, TGF-β1, FGF7, and ET-1 in BALF and cell culture supernatant were measured using human ELISA kits. ELISA assays were performed following the manufacturer’s protocols. ELISA kits for IL-1β, IL-6, TNF-α, TGF-β1, and FGF7 were purchased from Lianke Biotech (Hangzhou, China), and ELISA kits for ET-1 were purchased from Cusabio (Wuhan, China).

### 2.10 Western blot

Western blot was performed as previously described (17). Briefly, lung tissue samples and 16HBE cells were collected and lysed in RIPA lysis buffer (Wuhan Boster, China) supplemented with protease and phosphatase inhibitors (AR1178, Wuhan Boster, China) on ice. Protein concentrations were determined using the BCA assay (Beijing TransGen Biotech, China). Equivalent amounts of protein were loaded for each sample. The proteins were separated by SDS-PAGE and subsequently transferred onto PVDF membranes. After being blocked in 5% BSA, the membranes were incubated with primary antibodies at 4°C overnight and secondary antibodies at room temperature for 1 hour. After washing with 1×TBST (10× TBS buffer, 100 mL, distilled water 900 mL, 1 mL Tween 20), the blots were developed using ECL (Thermo Fisher, China), and images were processed by ChemiScope mini (Qinxiang, China). The band intensity was quantified using ImageJ. Antibodies used included β-actin (1:1000, 100166-MM10, Sino Biological); KGF (1:800-1:1000, ab131162, abcam); ADAM17 (1:800-1:1000, ab2051, abcam); p38 (1:1500-1:2500, ab170099, abcam); p-p38 (1:800-1:1000, ab47363, abcam); AKT (1:1000-1:1200, 4691S, CST); p-AKT (1:800-1:1000, ab192623, abcam); EGFR (1:1000, ab32077, abcam); p-EGFR (1:600-1:1000, 3777S, CST); ERK1/2 (1:1000, ab184699, abcam); p-ERK1/2 (1:800-1:1000, 9101S, CST); FGFR2 (1:1000, A19051, Abclonal); p-FGFR2 (1:500, PFGFR2-240AP, Thermo).

### 2.11 RT-qPCR

Total RNA was extracted using TRIzol reagent (15596026, Ambion, USA), and reverse transcription was performed using 5X All-In-One RT MasterMix (G492, Abm). Quantitative PCR was performed using QuantiNova SYBR Green Kit (208054, Kaijie) on QuantStudio™ 6 Flex (ABI, USA). Primer sequences are detailed in **Supplementary Tables 1**, **S2**.

### 2.12 Statistical analysis

All statistical data were shown as mean ± standard deviation (SD). Differences between the two groups were performed by unpaired t-tests, and multiple group comparisons were performed by one-way ANOVA. Differences were considered to be statistically significant at P<0.05. Statistical analysis was performed on GraphPad Prism 9.0 and SPSS 22.0.

## 3 Results

### 3.1 Aberrant expression of FGF7 in the lung tissue of COPD patients

We analyzed FGF7 expression in COPD and non-COPD individuals. Lung tissue samples (32 surgical specimens: 15 non-COPD control, 11 mild COPD, 5 moderate COPD, 1 severe COPD, detailed in **Supplementary Table 3**) and peripheral blood samples (71 blood samples: 20 non-COPD control, 11 mild COPD, 20 moderate COPD, 20 severe COPD) were collected for this study.

**Figure 1:**
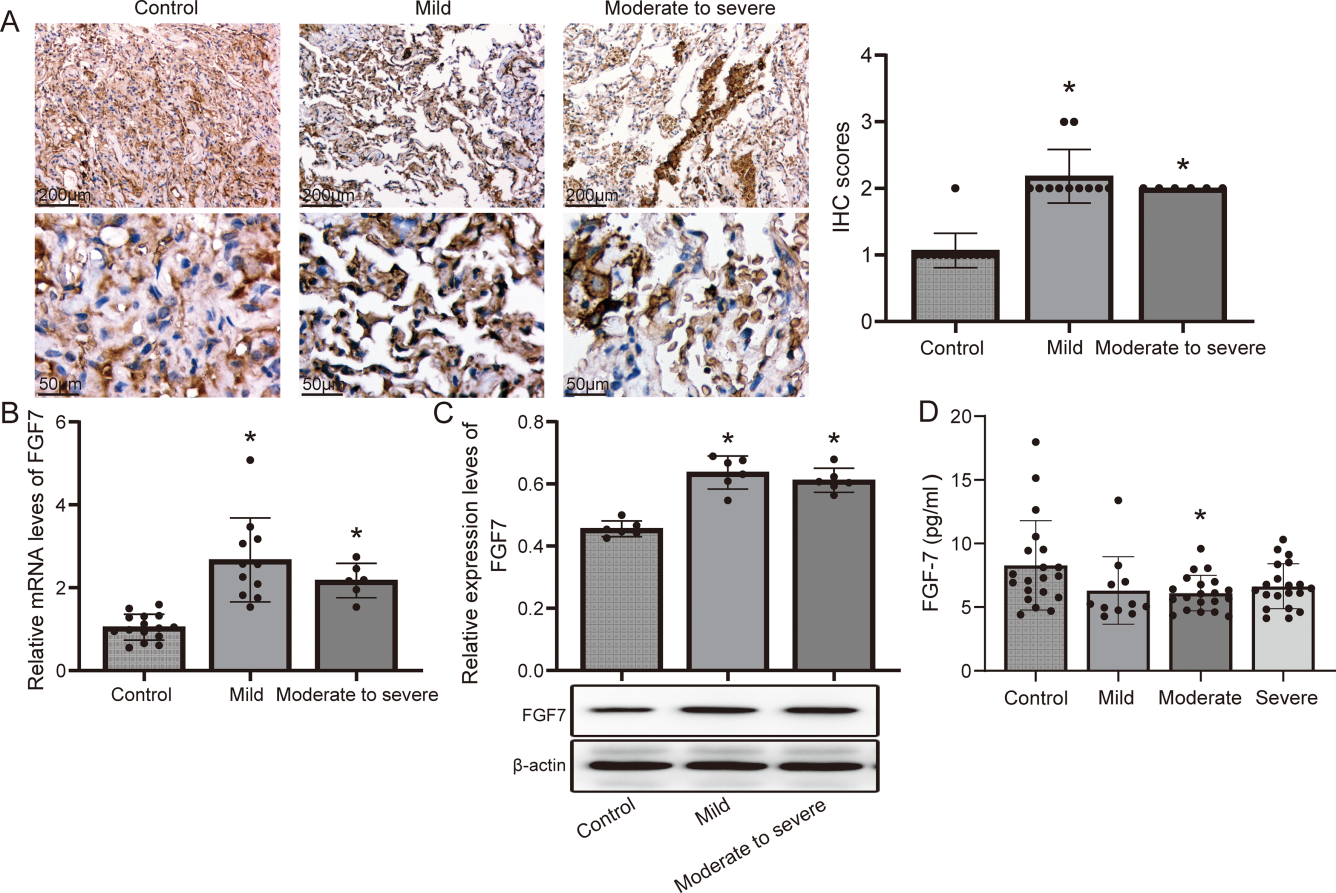
FGF7 is aberrantly expressed in lungs and peripheral blood of patients with COPD. **(A)** Representative IHC micrographs showing FGF7 localization in bronchial mucosal epithelium and alveolar epithelium from control and COPD lungs. Brown DAB signal indicates positive staining; nuclei counterstained with hematoxylin. Scale bars=200 μm and 50 μm. **(B)** RT-qPCR of FGF7 mRNA in lung tissue (normalized to reference gene; relative to control). n=32 surgical specimens (COPD: 11 mild, 5 moderate, 1 severe; controls: 15). **(C)** Western blot of FGF7 protein in lung tissue with β-actin as loading control (n=6 biological replicates). n=32 surgical specimens (COPD: 11 mild, 5 moderate, 1 severe; controls: 15). **(D)** Serum FGF7 quantified by ELISA in controls and COPD. n=71. *P < 0.05.

Immunohistochemical analysis of lung tissue specimens revealed that FGF7 was predominantly localized to the bronchial mucosal epithelium and alveolar epithelium (**Figure 1A**, **Supplementary Figure 1**). Semi-quantitative scoring indicated an elevated level of FGF7 in alveolar epithelial cells compared to airway epithelial cells in COPD cases (**Table 1**). Notably, increased levels of alveolar epithelial FGF7 protein were observed in mild and moderate-severe COPD patients compared to controls (**Supplementary Table 4**). After excluding samples with incomplete airway epithelium (2 mild, 1 moderate-severe COPD), FGF7 levels were also found to be elevated in airway epithelial cells of COPD patients (**Supplementary Table 5**). These results suggest an upregulation of FGF7 in both alveolar and airway epithelial cells in individuals with COPD.

**Table 1:** FGF7 comprehensive score of alveolar and airway epithelium in COPD patients (n=14).

	Weakly positive	Middle positive	Strongly positive	*P-value*
Alveolar epithelium	2	9	3	0.034
Airway epithelium	8	6	0	

Further validation through RT-qPCR and Western blot analyses confirmed an elevated expression of FGF7 in the lung tissues of COPD patients, although the differences across varying severities of COPD were not statistically significant (**Figures 1B, C**, **Supplementary Figure 2**). Notably, no upregulation of FGF7 was detected in the peripheral blood samples. In serum, FGF protein levels were either unchanged or slightly down-regulated in COPD patients (**Figure 1D**).

### 3.2 FGF7 overexpression attenuates CS-induced lung dysfunction, inflammation, and remodeling *in vivo*

**Figure 2:**
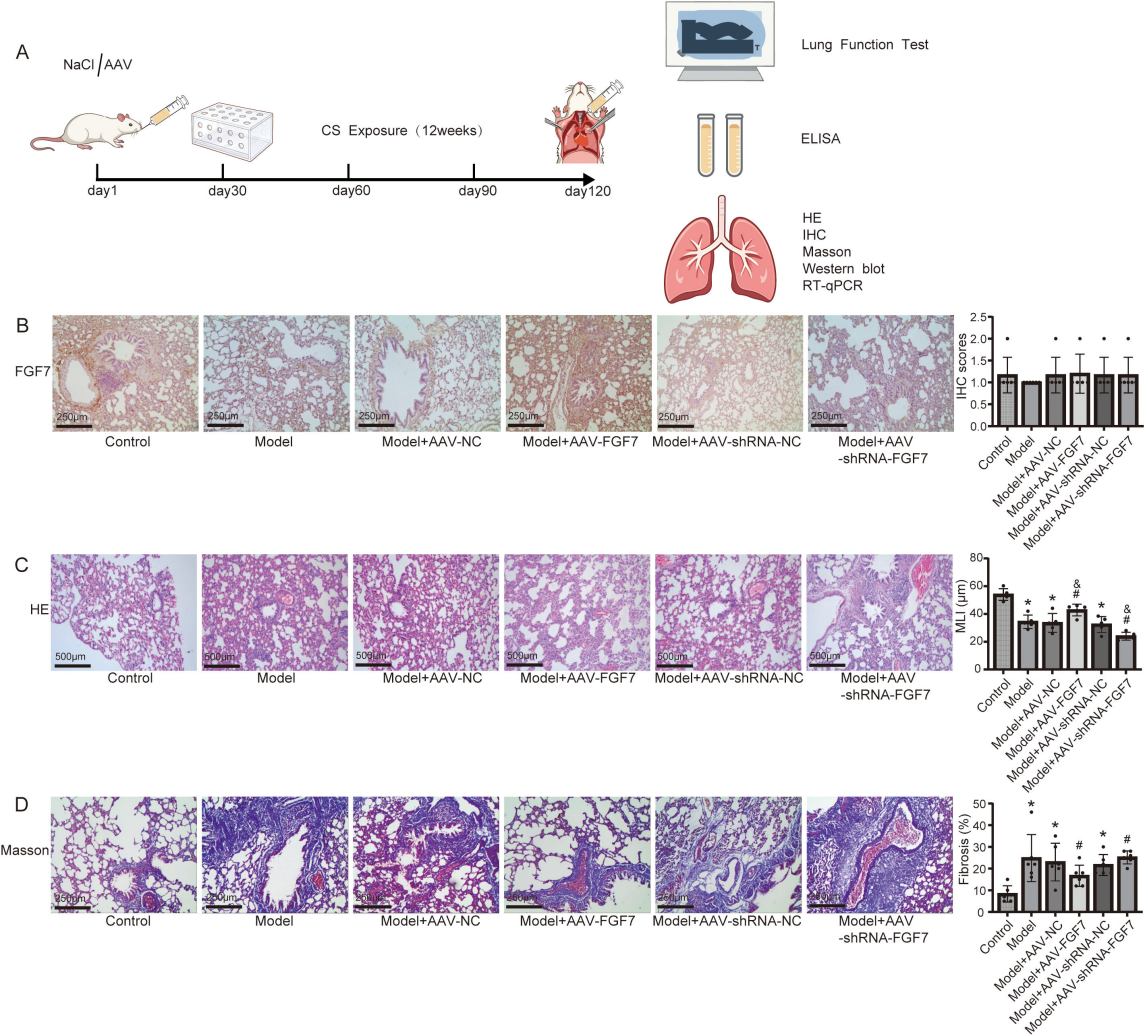
Histopathology and remodeling in the COPD rat model and the effect of FGF7 modulation. **(A)** Flow chart of animal experimental design. **(B)** IHC for FGF7 in rat lungs across groups. Scale bars=250 μm. **(C)** HE staining of airways and parenchyma from Control, Model, Model + AAV-NC, Model + AAV-FGF7, Model + AAV-shRNA-NC, and Model + AAV-shFGF7 groups. Scale bars=500 μm. Mean linear intercept (MLI) was determined using 5 random images from each of 3 H&E-stained lung sections per mouse (n=6 mice). **(D)** Masson’s trichrome staining highlighting airway wall collagen deposition (blue). Scale bars=250 μm. Quantitative fibrosis proportions are summarized in **Supplementary Table 6**. Compared to Control, * *P* < 0.05; Compared to Model, # *P* < 0.05; Compared to NC, & *P* < 0.05.

To investigate the effects of FGF7 on CS-induced COPD, we constructed a 12-week CS-exposure COPD rat model (**Figure 2A**). Through intratracheal administration of AAVs, we achieved FGF7 overexpression and knockdown in the model rats (**Supplementary Figure 3**). Immunohistochemistry confirmed the presence of FGF7 in lung sections of all groups, and further validated the overexpression and knockdown of FGF7 in the lung tissues using AAV-FGF7 and AAV-shFGF7, respectively (**Figure 2B**).

The histopathological assessment of lung tissues from model rats was conducted to evaluate disease severity. HE staining of lung sections revealed significant inflammation in both airways and lung parenchyma of COPD rats. The administration of AAV-FGF7 significantly attenuated inflammation and alleviated the destruction of alveolar architecture, while AAV-shFGF7 aggravated the histopathological changes (**Figure 2C**). Masson staining demonstrated increased collagen deposition in the airway wall post CS-exposure (**Figure 2D**). Administration of AAV-FGF7 mitigated collagen deposition, whereas AAV-shFGF7 enhanced it. The extent of pulmonary fibrosis was quantified based on Masson staining (**Supplementary Table 6**). Consistently, a significant reduction in pulmonary fibrosis was observed in rats with FGF7 overexpression, while a significant increase was noted in those with FGF7 knockdown. These histological findings suggest that FGF7 augmentation is associated with reduced inflammation and airway remodeling.

**Figure 3:**
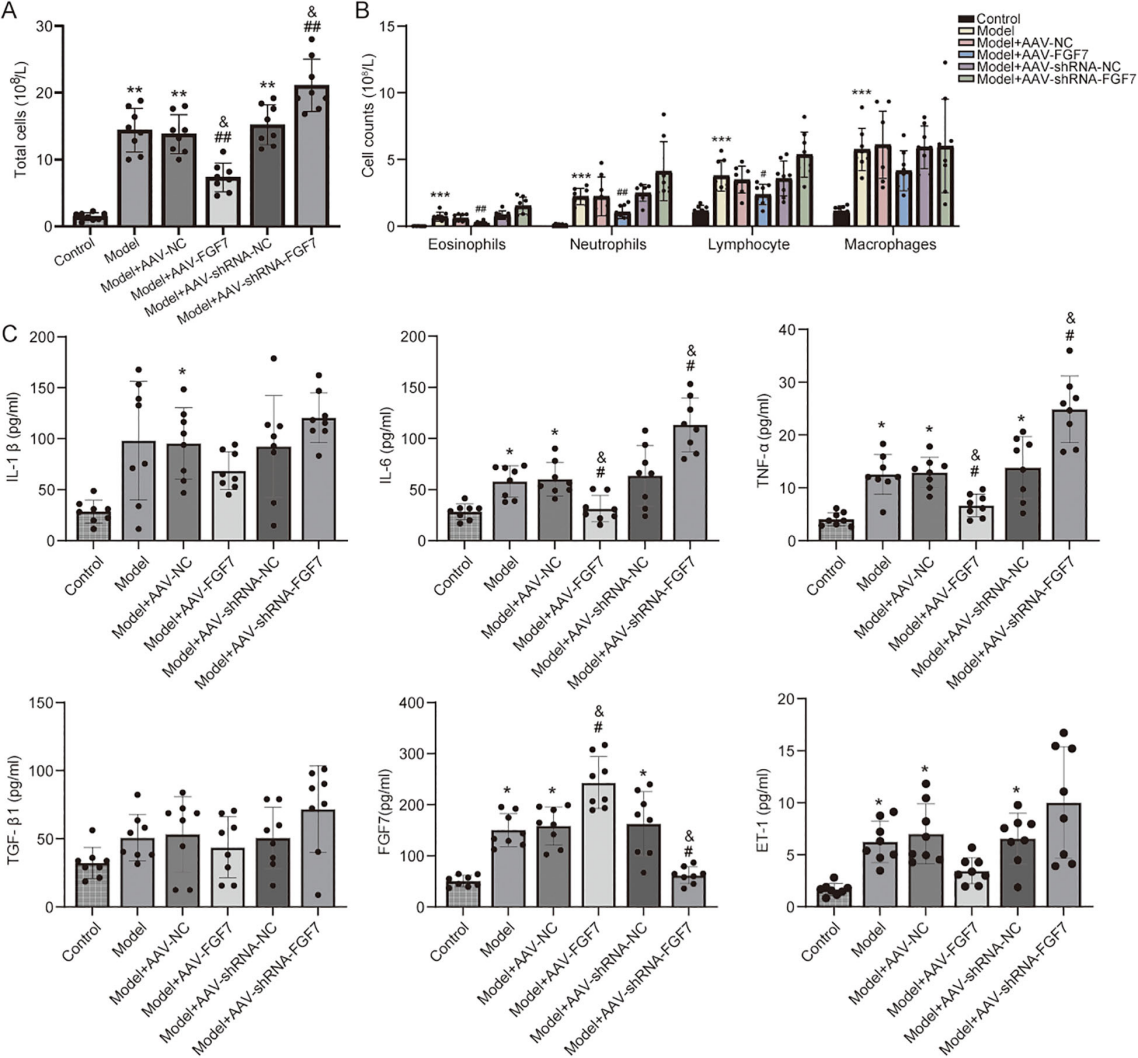
Airway inflammation assessed by BALF cellularity and cytokines in the COPD rat model with FGF7 modulation. **(A)** BALF total white cell counts across groups. **(B)** BALF differential counts (Eosinophils, Neutrophils, Lymphocyte, Macrophages). **(C)** BALF cytokines by ELISA. Compared to Control, * *P* < 0.05,** *P* < 0.01,***P <0.001; Compared to Model, # *P* < 0.05, ## *P* < 0.01, ###*P* < 0.001; Compared to NC, & *P* < 0.05.

Airway inflammation is often accompanied by an increase in total cell numbers within BALF. Here, we assessed the total cell count and the specific numbers of eosinophils, neutrophils, lymphocytes, and macrophages in BALF (**Figures 3A, B**). CS-exposure led to a significant increase in all four cell types. The ectopic expression of FGF7 resulted in a substantial decrease in eosinophils, neutrophils, and lymphocytes, although it did not affect the number of macrophages. In contrast, the knockdown of FGF7 caused a significant increase in the total cell count, with moderate but not statistically significant increases observed in eosinophils, neutrophils, and lymphocytes.

Inflammatory cytokine levels in BALF are also commonly used as markers of pulmonary inflammation. ELISA results showed that CS exposure significantly elevated the levels of IL-6, TNF-α, and ET-1, as well as FGF7, in BALF. The levels of IL-1β and TGF-β1 were also slightly increased, although these changes were not statistically significant (**Figure 3C**). Administration of AAV-FGF7 significantly reduced IL-6 and TNF-α levels. In contrast, AAV-mediated knockdown of FGF7 further elevated IL-6 and TNF-α levels in the BALF of CS-exposed rats. These findings suggested a suppressive role of FGF7 on the BALF inflammatory cytokine levels, especially impacting BALF levels of IL-6 and TNF-α.

Lung function assessments showed that CS exposure reduced ventilation parameters (MVb, PIFb, EF50), indicating obstructive dysfunction (**Table 2**). Overexpression of FGF7 (AAV-FGF7) improved these parameters compared to the model+AAV-NC groups, whereas knockdown of FGF7(AAV-shFGF7) worsened them, thereby demonstrating FGF7’s protective role against CS-induced obstruction.

**Table 2:** Lung function measurement data of rats.

Group	Function index							
	f(bpm)	TVb(mL)	MVb(mL/min)	PIFb(mL/sec)	PEFb(mL/sec)	Ti(sec)	Te(sec)	EF50(mL/sec)
Control	176.367±22.027	1.048±0.117	151.027±24.113	10.505±2.401	7.952±1.213	0.159±0.040	0.357±0.055	0.474±0.131
Model	133.038±25.741^a^	1.002±0.209	116.705±15.208^a^	7.653±1.314^a^	6.050±1.044^a^	0.193±0.024^a^	0.486±0.074^a^	0.319±0.123^a^
Model+AAV-NC	125.670±25.178	1.026±0.239	119.765±16.669	7.589±1.686	6.120±1.214	0.188±0.030	0.493±0.070	0.314±0.116
Model+AAV-FGF7	170.101±30.994^b^	0.967±0.123	148.459±14.860^b^	9.531±1.132^b^	6.514±1.176	0.168±0.044	0.401±0.063^b^	0.459±0.122^b^
Model+AAV-shRNA-NC	135.796±27.237	1.023±0.146	114.866±14.274	8.028±1.170	6.014±1.409	0.191±0.015	0.458±0.059	0.317±0.103
Model+AAV-shRNA-FGF7	122.063±24.950	1.074±0.179	80.163±15.210^c^	6.566±1.262^c^	5.159±1.549	0.227±0.030^c^	0.557±0.057^c^	0.269±0.062

Compared to control, ^a^ *P* < 0.05; Compared to Model+AAV-NC, ^b^ *P*<0.05; Compared to Model+AAV-shRNA-NC, ^c^ *P*<0.05.

f: respiratory rate; TVb: inspiratory volume; MVb: minute expiratory volume; PIFb: peak inspiratory flow rate; PEFb: peak expiratory flow rate; Ti: inspiratory time; Te: expiratory time; EF50: exhalation flow rate

In conclusion, the overexpression of FGF7 effectively alleviated lung injury across various parameters, whereas its knockdown intensified the symptoms. These results confirmed FGF7’s protective role against CS-induced airway inflammation and remodeling in COPD models.

### 3.3 AAV-FGF7 activates the ADAM17-EGFR-ERK1/2 axis

**Figure 4:**
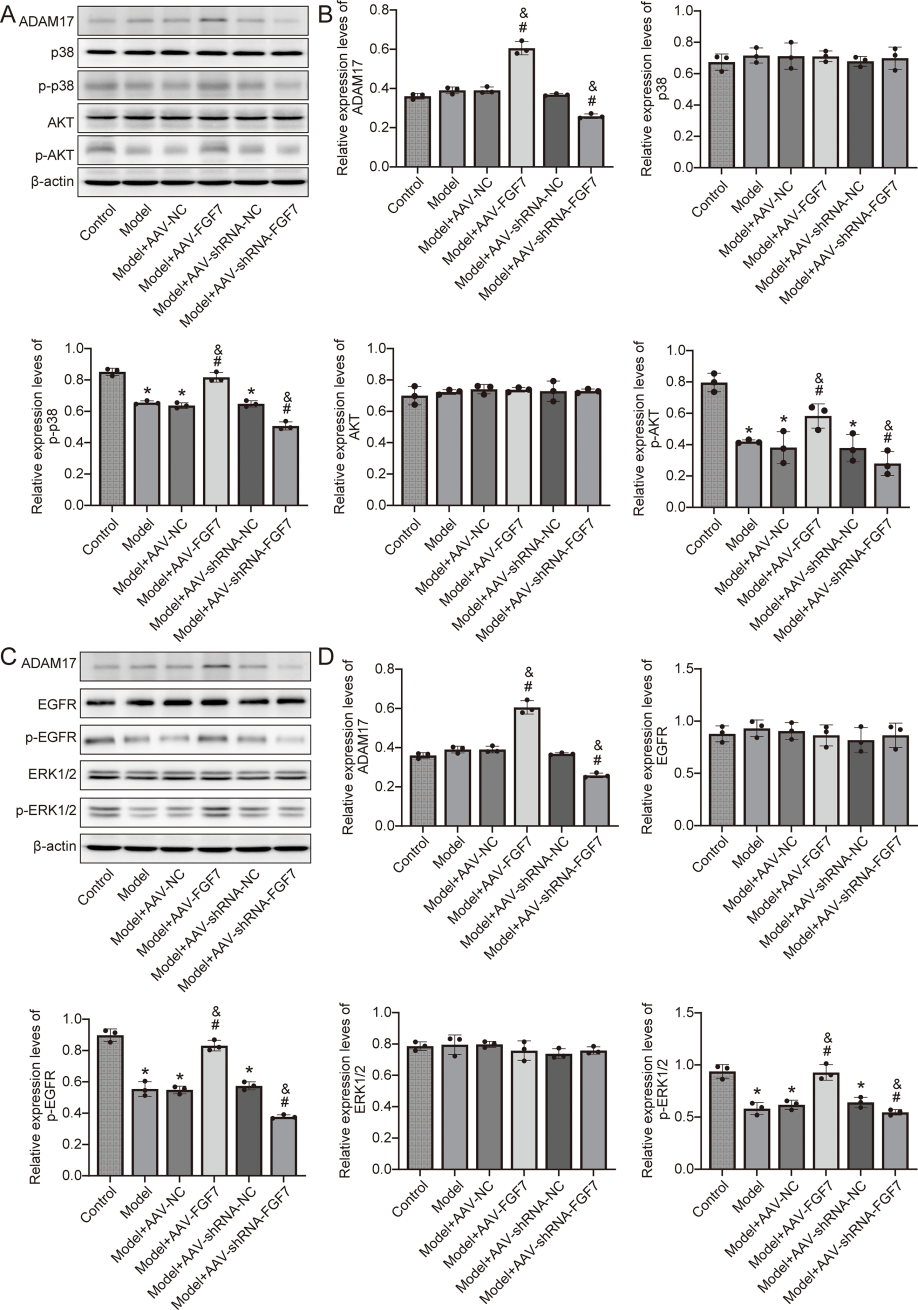
The activation of ADAM17 in rat lung tissues. **(A, B)** Representative Western blots and quantification of ADAM17, p-p38/p38, and p-AKT/AKT in lung homogenates from Control, Model (CS), Model + AAV-NC, Model + AAV-FGF7, Model + AAV-shRNA-NC, and Model + AAV-shFGF7 (n=3 biological replicates). **(C, D)** Representative Western blots and quantification of ADAM17, p-EGFR/EGFR, and p-ERK1/2/ERK1/2 in lung homogenates from Control, Model (CS), Model + AAV-NC, Model + AAV-FGF7, Model + AAV-shRNA-NC, and Model + AAV-shFGF7 (n=3 biological replicates). Compared to Control, *P < 0.05; Compared to Model, #*P* < 0.05; Compared to NC, & *P* < 0.05.

Given FGF7’s protective effects against airway inflammation and remodeling, we explored the downstream mechanisms of FGF7 in COPD rat models. FGF7, also known as keratinocyte growth factor, has been documented to activate the extracellular signal-regulated kinases ERK1/2 pathway, thereby regulating the proliferation and migration of epithelial cells (18, 19). ADAM17, a membrane-anchored metalloproteinase, is recognized as a central component in this signaling pathway, with its release from the nuclear membrane necessitating the activation of p38 and AKT (19). Therefore, we first evaluated the activation of ADAM17 in rat lung tissues. Western blot analyses demonstrated that AAV-FGF7 upregulated the cellular level of ADAM17, p-p38, and p-AKT1, whereas AAV-shFGF7 inhibited the activation of p38, AKT1, and ADAM17 (**Figures 4A, B**).

In epithelial cells, ADAM17 is known to activate EGFR and its downstream signaling cascades, thereby promoting cell proliferation and wound healing (19, 20). Specifically, in lung epithelial cells, it has been reported that cigarette smoke exposure enhances ADAM17-mediated activation of the EGFR-ERK1/2 pathway (21). Thus, we further investigated the influence of FGF7 levels on the activation of EGFR-ERK1/2 signaling. As anticipated, the introduction of AAV-FGF7 led to enhanced activation of EGFR and ERK1/2. Conversely, AAV-shFGF7 attenuated the phosphorylation of EGFR and ERK1/2 (**Figures 4C, D**). Collectively, these findings indicate that FGF7 promotes epithelial repair through ADAM17-dependent EGFR-ERK1/2 signaling.

### 3.4 Recombinant FGF7 reverses CSE-induced inhibition of epithelial proliferation and migration

**Figure 5:**
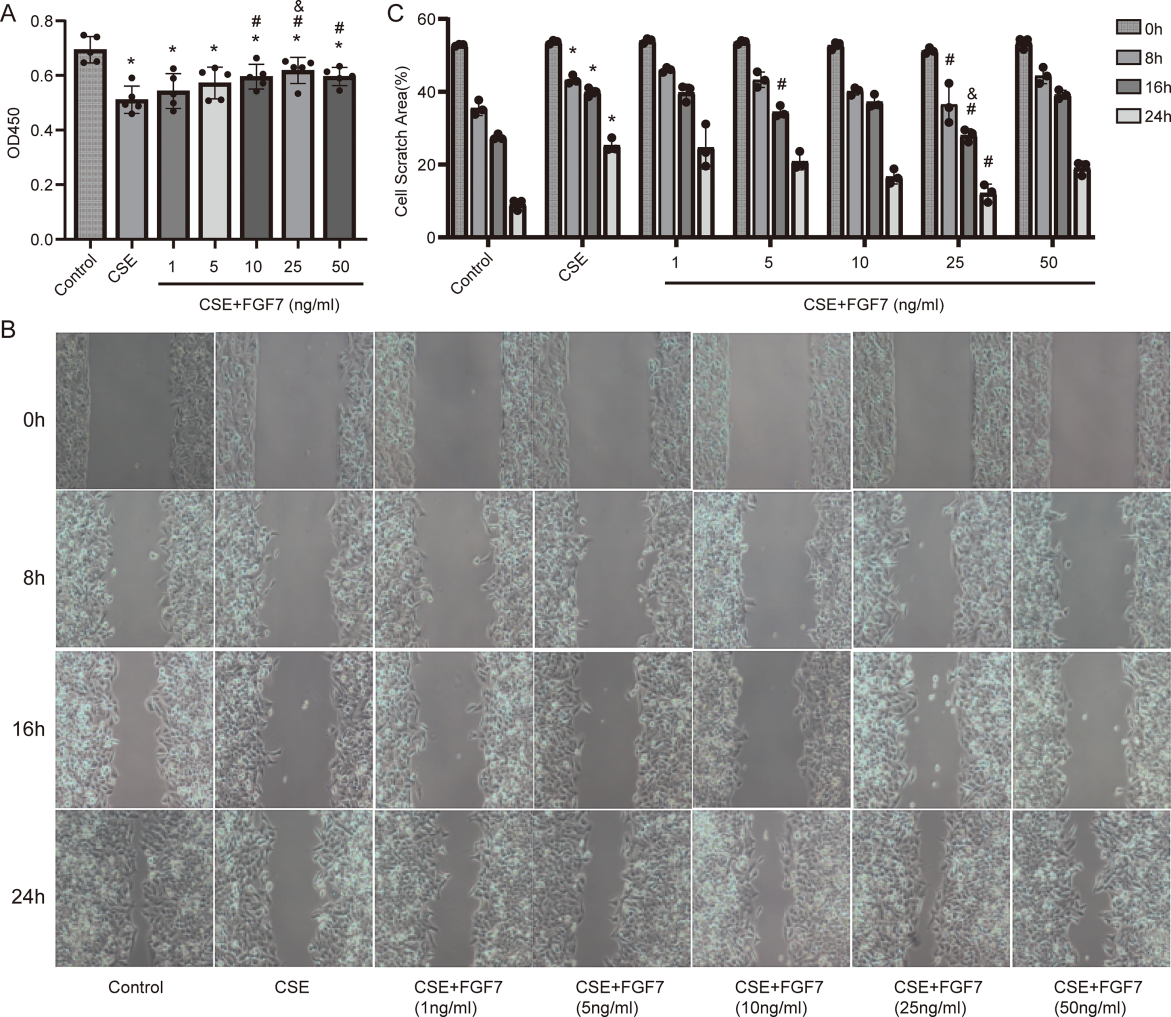
rFGF7 reverses CSE-induced suppression of epithelial proliferation and migration in 16HBE cells. **(A)** CCK-8 viability under graded rFGF7 (0–50 ng/mL) with 5% CSE for 36 h. **(B)** Scratch-wound assay representative images (0 and 24 h) for Control, CSE (5%), CSE + rFGF7 (1–50 ng/mL). Scale bars=200 μm. **(C)** Quantified wound closure (%) over time. Compared to Control, *P < 0.05; Compared to CSE, #*P* < 0.05; Compared to CSE+FGF7 (1 ng/ml), & *P* < 0.05.

To further elucidate the role of FGF7 in CS-induced COPD, 16HBE cells were treated with CSE to stimulate smoking condition *in vitro*. Preliminary CCK-8 assays were conducted on 16HBE cells exposed to 1-20% CSE with a time course of 12, 24, and 36 hours. Treatment conditions of 5% CSE for 36 hours were used in the following analysis (**Supplementary Figure 4**). Various concentrations of recombinant FGF7 (rFGF7) were employed to mitigate CSE-induced cell death. Recombinant FGF7 restored 16HBE cell proliferation in a dose-dependent manner, with 10 ng/mL significantly enhancing cell viability compared to CSE treatment alone, and 25 ng/mL exhibiting the most pronounced effect (**Figure 5A**). Scratch assays demonstrated that rFGF7 also restored cell migration, with 5 ng/mL showing significant improvement compared to CSE, and 25 ng/mL being the most potent at 16 hours (**Figures 5B, C**). Collectively, these findings support the notion that FGF7 counteracts CSE-induced inhibition of epithelial cell growth and migration, aligning with its reparative role in the airway epithelium.

### 3.5 Recombinant FGF7 inhibits inflammation and activates ADAM17-ERK1/2 signaling

**Figure 6:**
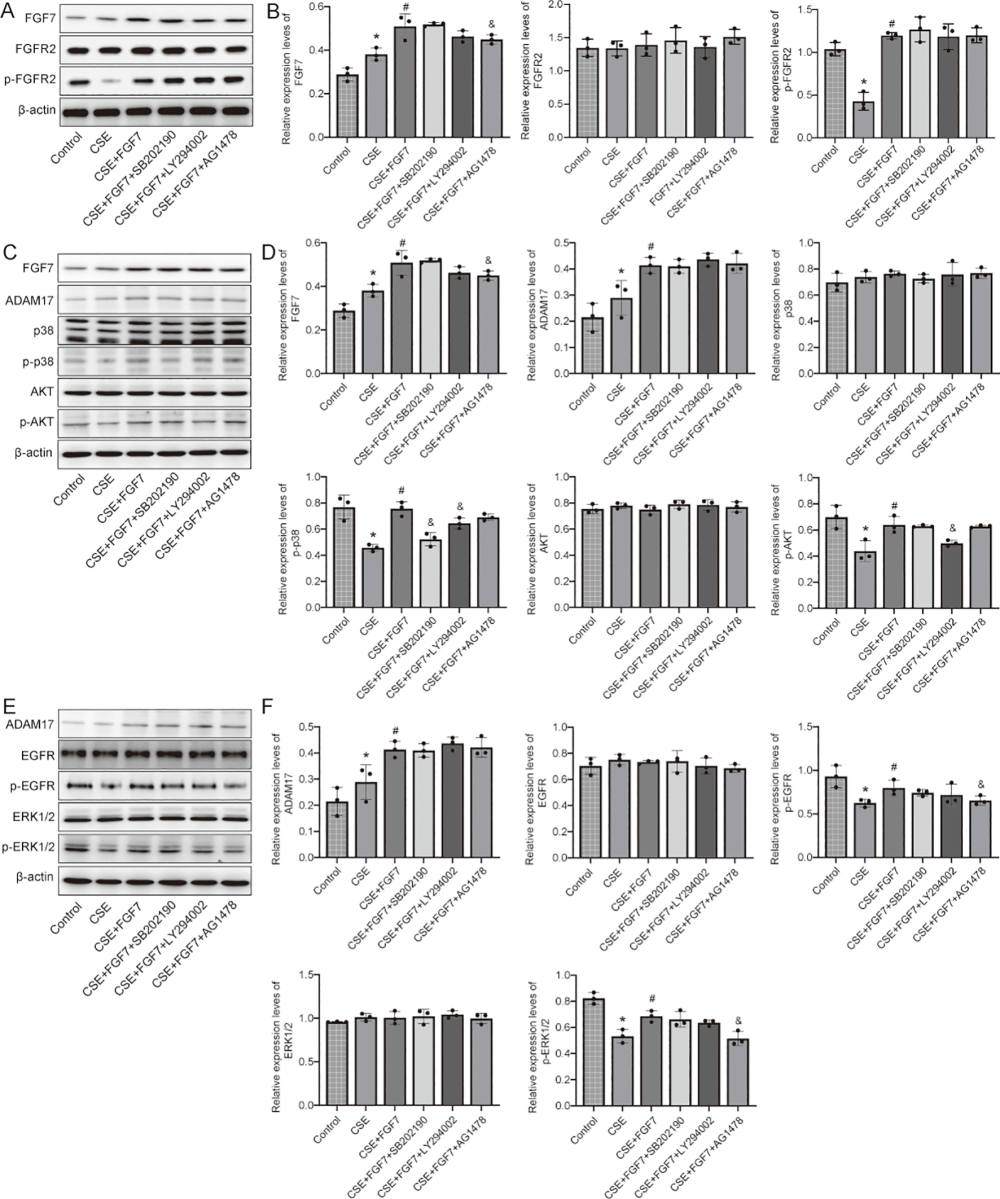
The effect of FGF7 on the ADAM17-EGFR-ERK1/2 signaling pathway in 16HBE cells. **(A, B)** Representative Western blots and densitometry for FGF7 and p-FGFR2/FGFR2 under the following conditions: Control, CSE (5%), CSE + rFGF7, and CSE + rFGF7 with SB202190 (p38 inhibitor, 10 μM, 1 h pretreat), LY294002 (PI3K inhibitor, 10 μM, 1 h), or AG1478 (EGFR inhibitor, 10 μM, 1 h) (n=3 biological replicates). **(C, D)** Representative Western blots and densitometry for FGF7, ADAM17, p-p38/p38, and p-AKT/AKT under the following conditions: Control, CSE (5%), CSE + rFGF7, and CSE + rFGF7 with SB202190 (p38 inhibitor, 10 μM, 1 h pretreat), LY294002 (PI3K inhibitor, 10 μM, 1 h), or AG1478 (EGFR inhibitor, 10 μM, 1 h) (n=3 biological replicates). **(E, F)** Representative Western blots and densitometry for ADAM17, p-EGFR/EGFR, and p-ERK1/2/ERK1/2 under the following conditions: Control, CSE (5%), CSE + rFGF7, and CSE + rFGF7 with SB202190 (p38 inhibitor, 10 μM, 1 h pretreat), LY294002 (PI3K inhibitor, 10 μM, 1 h), or AG1478 (EGFR inhibitor, 10 μM, 1 h) (n=3 biological replicates). Compared to Control, *P < 0.05; Compared to CSE, #*P* < 0.05; Compared to CSE+FGF7, & *P* < 0.05.

To validate that FGF7 also activates the ADAM17-EGFR-ERK1/2 signaling axis in human airway epithelial cells, Western blots were performed on 16HBE cells treated with CSE and rFGF7. Consistent with the results observed in the rat model, the data demonstrated that CSE treatment resulted in decreased level of cellular ADAM17 and reduced phosphorylation of p38 and AKT, as well as FGFR2, which is known as the primary high-affinity receptor for FGF7 (22) (**Figures 6A–D**). In contrast, treatment with rFGF7 restored these phosphorylations and upregulated ADAM17 levels. Accordingly, the activation of downstream signaling molecules EGFR and ERK1/2 was enhanced by rFGF7 (**Figures 6E, F**). The application of specific inhibitors—SB202190, LY29400, and AG1478, targeting p38, PI3K, and EGFR, respectively—partially mitigates the effects of rFGF7 on the corresponding phosphorylation events, without altering the expression or activation status of FGF7 and FGFR2. This observation further implies that FGF7-FGFR2 probably functions upstream of the ADAM17-ERK1/2 signaling pathway (**Figure 6**).

**Figure 7:**
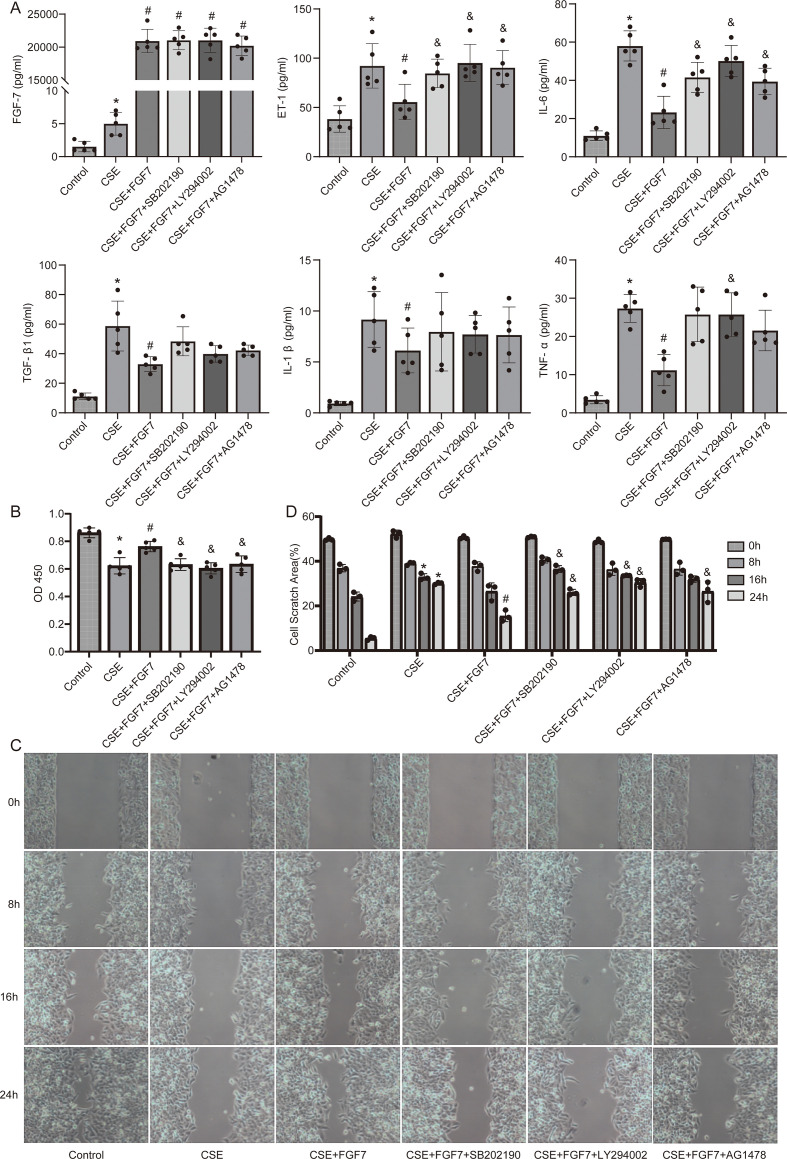
FGF7 suppresses epithelial cytokine outputs and its pro-repair effects require p38, PI3K, and EGFR signaling. **(A)** ELISA of ET-1, IL-6, TGF-β1, IL-1β, and TNF-α in 16HBE supernatants. CSE elevates cytokines; rFGF7 significantly reduces them versus CSE alone. **(B)** CCK-8 proliferation under Control, CSE, CSE + rFGF7, and CSE + rFGF7 with SB202190, LY294002, or AG1478. Inhibitors attenuate rFGF7-mediated rescue. **(C, D)** Scratch-wound migration quantification under the same conditions as **(B)**. Compared to Control, *P < 0.05; Compared to CSE, #*P* < 0.05; Compared to CSE+FGF7, & *P* < 0.05.

Additionally, CSE exposure led to an increase in cytokines in the cell culture supernatant, including ET-1, IL-6, TGF-β1, IL-1β, and TNF-α. Treatment with rFGF7 significantly reduced the levels of ET-1, IL-6 and TNF-α compared to CSE exposure alone (**Figure 7A**). Inhibition of p38, PI3K, or EGFR attenuated the anti-inflammatory and reparative effects of rFGF7 (**Figures 7B–D**), further confirming the essential role of these signaling nodes in mediating the action of FGF7.

## 4 Discussion

Our findings in COPD patient tissues, rat models, and CSE-injured airway epithelial cells consistently identified FGF7 as a lung-specific, epithelial-protective factor in COPD. In COPD patients, FGF7 expression is upregulated in pulmonary epithelial cells but downregulated in circulation, suggesting localized, potentially consumptive epithelial responses rather than a systemic increase. This observation aligns with the known paracrine role of FGF7 in lung repair and regeneration (8, 23). The absence of distinct severity gradients across COPD subgroups was attributed to limited sample sizes and the cross-sectional nature of the study. Nonetheless, the consistent upregulation observed suggests compensatory repair mechanisms that may become insufficient as the disease progresses. We proposed that upregulation of FGF7 in the early stage of COPD facilitates the repair of lung injury, whereas the compensatory capacity may diminish in advanced stages.

As a member of the fibroblast growth factor family, FGF7 canonically signals via FGFR2 to activate intracellular cascades, prominently the Ras-ERK/MAPK pathway, which controls proliferation and differentiation in multiple cell types (22, 24, 25). In this study, our data support an FGF7-ADAM17-EGFR-ERK1/2 signaling pathway in airway epithelial cells, with p38 and PI3K/AKT serving as cooperative nodes. *In vitro* experiments revealed that CSE inhibited these kinases, while rFGF7 restored their activity and upregulated ADAM17. The selective inhibition of p38, PI3K, or EGFR reduced the reparative and anti-inflammatory effects of FGF7 without altering the cellular level or activation status of FGF7 and FGFR2, indicating that FGF7-FGFR2 acts upstream of these signaling nodes to conduct its anti-inflammatory function. The use of shFGF7 resulted in reduced ERK1/2 activity and increased inflammation, further substantiating the reliance of this signaling axis on endogenous FGF7. *In vivo*, administration of AAV-FGF7 led to upregulation of ADAM17 and phosphorylation of EGFR/ERK1/2, accompanied by parallel changes in p-p38 and p-AKT changes, whereas AAV-shFGF7 attenuated these signals. These findings are consistent with previous evidence indicating that ADAM17-mediated EGFR transactivation promotes epithelial repair (19) and that FGF7/KGFR signaling confers epithelial protection (18, 23).

The pathogenesis of COPD is characterized by chronic inflammation, oxidative stress, CS-induced barrier dysfunction, and dysregulation of immune-epithelial communication (26, 27). In alignment with these mechanisms. our model demonstrated that exposure to CS/CSE resulted in elevated BALF cell counts and increased levels of cytokines such as IL-6, TNF-α, IL-1β, TGF-β1, and ET-1. These effects were mitigated by FGF7 and exacerbated in its absence. Previous studies have indicated that oxidants derived from CS recruit macrophages and neutrophils, causing damage to endothelial and epithelial cells (28). Our findings suggest that FGF7 operates upstream of the ADAM17-EGFR-ERK1/2 repair pathway, which suppresses cytokine production while enhancing epithelial growth and motility during CS exposure. Notably, in this study, ectopic FGF7 was administered concurrently with CS exposure, suggesting a preventive role for FGF7 in CS-induced COPD. To further explore its translational applications, it is necessary to examine whether ectopic administration of FGF7 can reverse the epithelial damage and airway remodeling already present in COPD patients. Although our study provides valuable insights into the protective effects of FGF7 against CS-induced injury, the *in vitro* mechanistic experiments were conducted exclusively using the 16HBE cell line. While 16HBE cells serve as a well-established and robust model for investigating airway epithelial cell signaling and barrier function, their status as an immortalized cell line may not fully replicate the complex physiological and pathological responses of primary human airway tissues. Therefore, future studies utilizing primary human bronchial epithelial cells are warranted to further validate the physiological and translational significance of the FGF7-ADAM17-EGFR-ERK1/2 signaling axis identified in this research.

From the perspective of epithelial repair, FGF7 inhibits apoptosis, promotes proliferation and migration, and improves barrier function across multiple injury models. In alveolar type II cells, FGF7 confers protection through the PI3K/Akt/mTOR pathway (29); in models of neonatal hyperoxia and fibrosis, it synergizes with mesenchymal stem cells (MSCs) to exert anti-fibrotic effects (30); in ischemia-reperfusion on injury, it activates Nrf2 to reduce cell death (31); and it play a role in regulating surfactant homeostasis (32). These findings are consistent with our observations in 16HBE cells, where rFGF7 was shown to restore cell viability and migration while reducing inflammation, thereby underscoring its anti-inflammatory and anti-remodeling potential. The knockdown of FGF7 resulted in decreased ERK1/2 activity and heightened inflammation, aligning with the exacerbation of pathological conditions in the absence of FGF7. Studies on skin wounds and other epithelial tissues have demonstrated that interventions involving FGF7/KGFR modulate healing processes through TGF-β1/Smad or differentiation-related receptors (33, 34), further corroborating FGF7’s roles as a broadly protective epithelial factor.

Noticeably, in the lung tissue of COPD patients, there is a marked local increase in FGF7, while its serum levels are slightly decreased. This observation suggests an attempt by the lung to initiate a localized epithelial response. In this context, the elevation of FGF7 is likely a compensatory protective mechanism against cigarette smoke-induced inflammation and epithelial injury. This is supported by our finding that FGF7 overexpression mitigated, whereas FGF7 knockdown exacerbated, smoke-induced inflammation and airway remodeling. It is important to note that FGF7 intervention in this study was introduced during cigarette smoke exposure, prior to the full establishment of COPD-like pathology, indicating that FGF7 primarily plays a role in early or injury-limiting responses. Further research is necessary to assess the potential of FGF7 in reversing established COPD.

## 5 Conclusion

This study identifies FGF7 as a locally induced epithelial protective factor that mitigates inflammation and facilitates repair following smoke-induced injury through ADAM17-dependent EGFR/ERK1/2 signaling. The tissue-specific induction and improved functional recovery underscore the potential of the FGF7 signaling axis as a promising target for the prevention of CS-induced airway inflammation and epithelial damage in COPD.

## Data Availability

The datasets presented in this study can be found in online repositories. The names of the repository/repositories and accession number(s) can be found in the article/**Supplementary Material**.

## Supplementary material

Supplementary Figure 1 The IHC staining images for all 32 specimens.

Supplementary Figure 2 The Western blot results of surgical specimens.

Supplementary Figure 3 Validation of FGF7 knockout and overexpression. **(A)** RT-qPCR of FGF7 mRNA in lung tissue of rat. **(B)** Western blot of FGF7 protein in lung tissue of rat with β-actin as loading control (n=3 biological replicates). Compared to Control, * *P* < 0.05; Compared to Model, # *P* < 0.05; Compared to NC, & *P* < 0.05.

Supplementary Figure 4 Determination of the effective dose of CSE intervention on cells.

Supplementary Table 1 Sequence of the primers for RT-qPCR (Human).

Supplementary Table 2 Sequence of the primers for RT-qPCR (Rat).

Supplementary Table 3 Baseline characteristics of the study participants.

Supplementary Table 4 Semi-quantitative IHC scores of alveolar epithelium FGF7 in controls and COPD subgroups. (n=32).

Supplementary Table 5 Semi-quantitative IHC scores of airway epithelium FGF7 in controls and COPD subgroups. (n=29).

Supplementary Table 6 Quantification of airway wall fibrosis by Masson’s trichrome in experimental rat groups.
