# Supplementary figures and images for: FGF7 mitigates airway inflammation and epithelial injury in cigarette smoke-induced COPD model
Source: Front Immunol. 2026 Jun 8;17:1815550. doi: 10.3389/fimmu.2026.1815550 (PMC13283814; doi:10.3389/fimmu.2026.1815550)

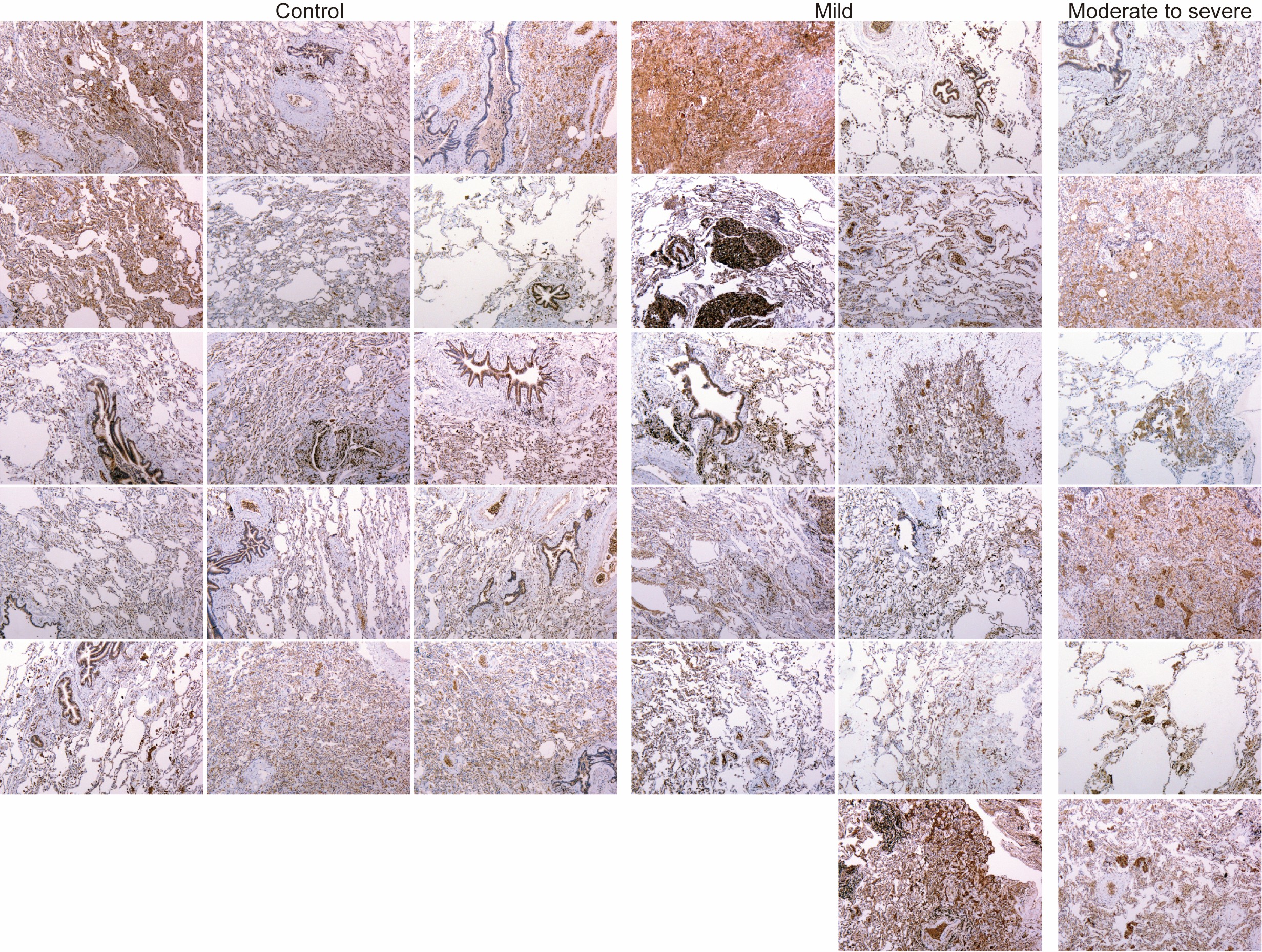

Supplement: Supplementary Figure 1 — The IHC staining images for all 32 specimens. [file Image1.jpeg]

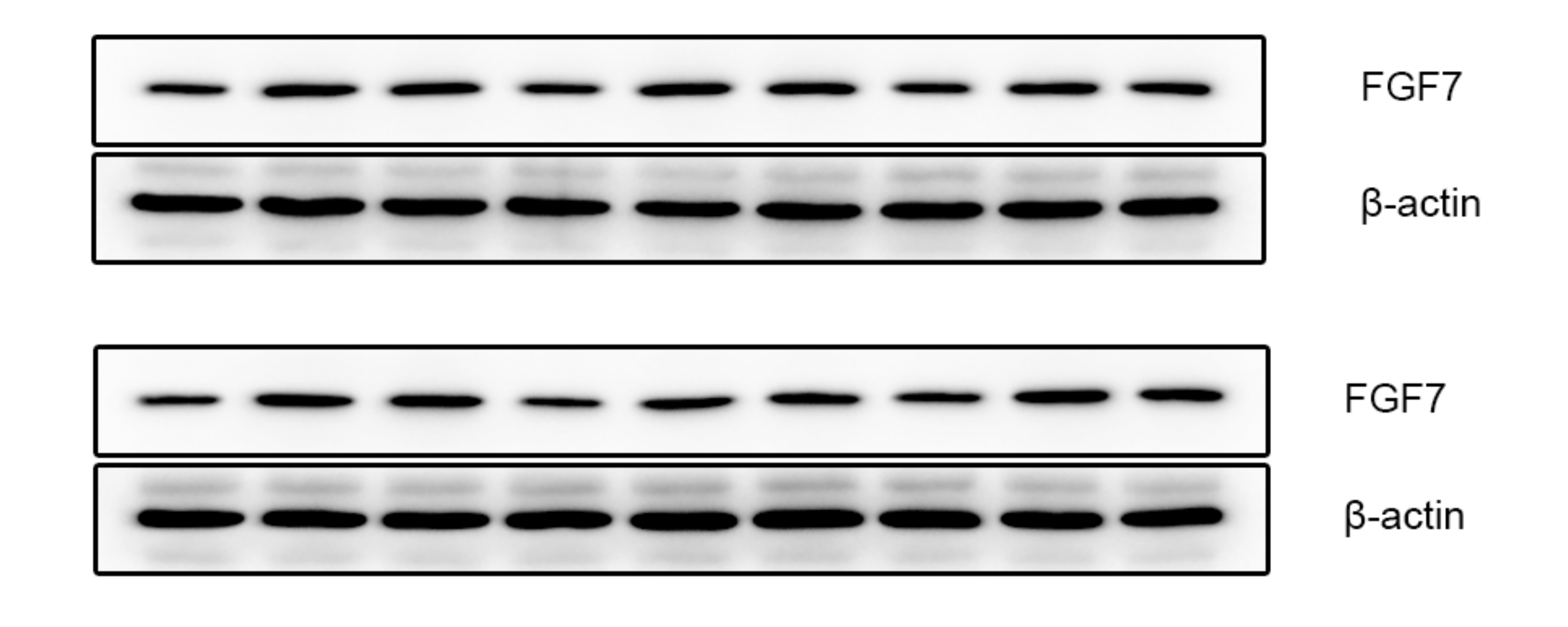

Supplement: Supplementary Figure 2 — The Western blot results of surgical specimens. [file Image2.jpeg]

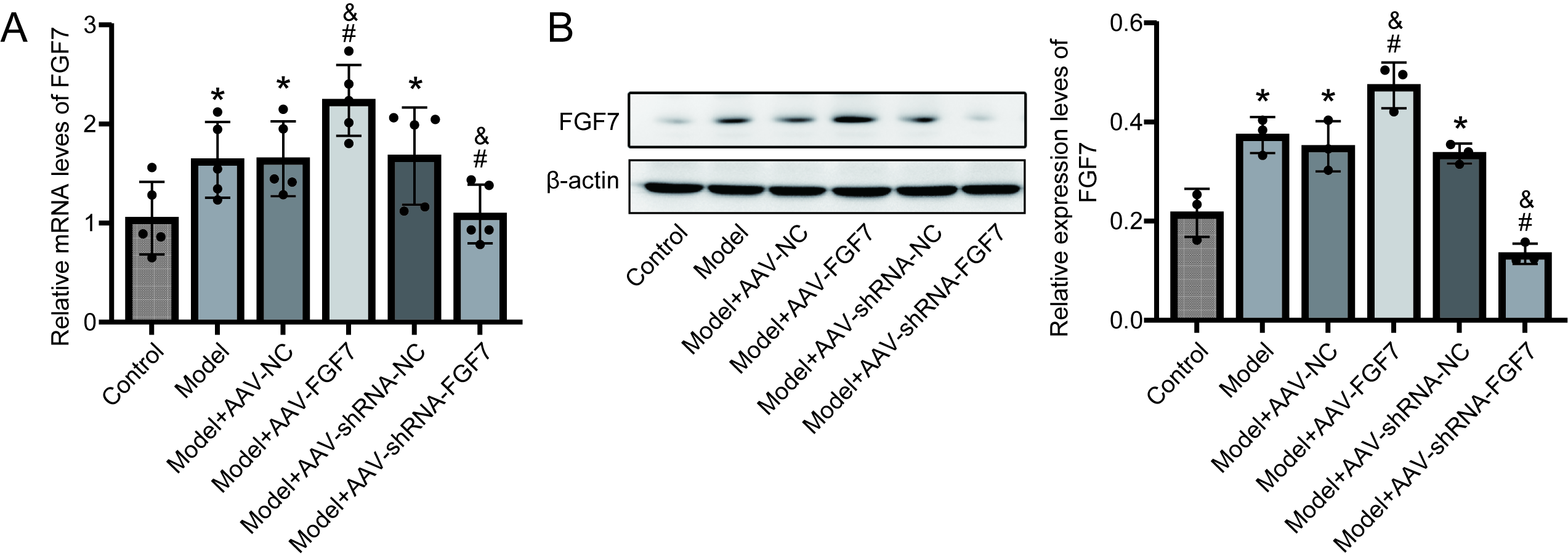

Supplement: Supplementary Figure 3 — Validation of FGF7 knockout and overexpression. (A) RT-qPCR of FGF7 mRNA in lung tissue of rat. (B) Western blot of FGF7 protein in lung tissue of rat with β-actin as loading control (n=3 biological replicates). Compared to Control, * P < 0.05; Compared to Model, # P < 0.05; Compared to NC, & P < 0.05. [file Image3.tif]

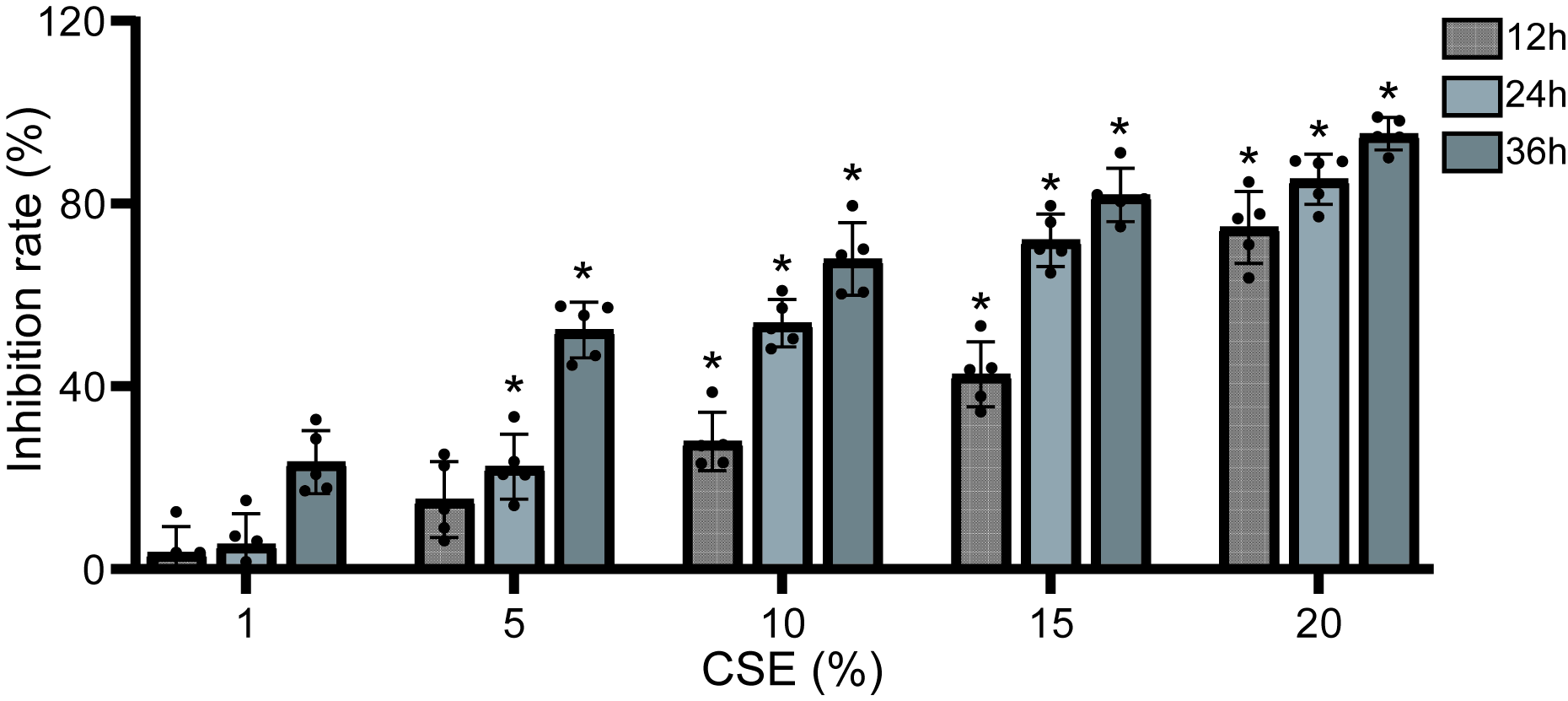

Supplement: Supplementary Figure 4 — Determination of the effective dose of CSE intervention on cells. [file Image4.tif]
