# Supplementary material for: FGF7 mitigates airway inflammation and epithelial injury in cigarette smoke-induced COPD model
Source: Front Immunol. 2026 Jun 8;17:1815550. doi: 10.3389/fimmu.2026.1815550 (PMC13283814; doi:10.3389/fimmu.2026.1815550)
Supplement: Supplementary Table 1 — Sequence of the primers for RT-qPCR (Human). [file Table1.docx]

**Table S1.** Sequence of the primers for RT-qPCR (Human).

| Gene | Sequence (5' to 3') | Primer size |
| --- | --- | --- |
| FGF7-F | ATCCTTCTGCCTGTTGATTT | 124bp |
| FGF7-R | ATTCCATGTCTGTTGTCTGC |  |
| ADAM17-F | CTGAGCCGGCCTTTGGTAA | 149bp |
| ADAM17-R | CGGAAAACTGCTCACATCGG |  |
| ERK1-F | CACCCTGGAAGCCATGAGAG | 127bp |
| ERK1-R | CCGCAGGATCTGGTAGAGGA |  |
| ERK2-F | CGTGTTGCAGATCCAGACCA | 143bp |
| ERK2-R | GCCAGAATGCAGCCTACAGA |  |
| GAPDH-F | TGTTGCCATCAATGACCCCTT | 250bp |
| GAPDH-R | CTCCACGACGTACTCAGCG |  |

**Table S2.** Sequence of the primers for RT-qPCR (Rat).

|  | Sequence (5' to 3') | Primer size |
| --- | --- | --- |
| FGF7 F | AGGAGACTGTTCTGTCGCAC | 144bp |
| FGF7 R | TTCCACCCCTTTGATTGCCA |  |
| GAPDH_F | CAGGGCTGCCTTCTCTTGTG | 172bp |
| GAPDH_R | GATGGTGATGGGTTTCCCGT |  |

**Table S3.** Baseline characteristics of the study participants

| Variable | Control group | Case Group |
| --- | --- | --- |
| Age, years | 53.5±10.3 | 59.9±9.5 |
| Sex(Male/Female) | 11/4 | 5/12 |
| Smoking history,% | 13.3% | 52.9% |
| Lung function, n |  |  |
| Normal | 15 |  |
| Mild |  | 11 |
| Moderate |  | 5 |
| Severe |  | 1 |

**Table S4.** Semi-quantitative IHC scores of alveolar epithelium FGF7 in controls and COPD subgroups (n=32)

| Group | Weakly positive | Middle positive | strongly positive | *P-value* |
| --- | --- | --- | --- | --- |
| Control | 14 | 1 | 0 | *P_1_*＜0.001 |
| Mild | 0 | 7 | 4 | *P_2_*=0.011 |
| Moderate to severe | 2 | 4 | 0 | *P_3_*=0.063 |

*P*_1_: Mild group and control group; *P_2_*: Moderate to severe group and control group; *P_3_*: Mild group and moderate to severe group.

**Table S5.** Semi-quantitative IHC scores of airway epithelium FGF7 in controls and COPD subgroups (n=29)

| Group | Weakly positive | Middle positive | *P-value* |
| --- | --- | --- | --- |
| Control | 14 | 1 | 0.035 |
| COPD | 8 | 6 |  |

**Table S6.** Quantification of airway wall fibrosis by Masson’s trichrome in experimental rat groups.

| Group | Proportion of pulmonary fibrosis（%） |
| --- | --- |
| Control | 8.333±3.777 |
| Model | 24.833±10.852^△^ |
| Model+AAV-NC | 23.000±8.672^△^ |
| Model+AAV-FGF7 | 16.667±4.885^△▲^ |
| Model+AAV-shRNA-NC | 21.667±4.803^△^ |
| Model+AAV-shRNA-FGF7 | 25.167±2.994^△▼^ |
| *F* | 5.720 |
| *P* | 0.001 |

Compared to control, △*P* < 0.01; Compared to model, ▲*P* < 0.01; Compared to AAV-NC, ▽ *P* < 0.05; Compared to AAV-FGF7, ▼*P*<0.05.
